# Age-Dependent Neuropsychiatric Symptoms in the NF-κB/c-Rel Knockout Mouse Model of Parkinson’s Disease

**DOI:** 10.3389/fnbeh.2022.831664

**Published:** 2022-03-11

**Authors:** Edoardo Parrella, Federico Del Gallo, Vanessa Porrini, Cristina Gussago, Marina Benarese, Paolo Francesco Fabene, Marina Pizzi

**Affiliations:** ^1^Division of Pharmacology, Department of Molecular and Translational Medicine, University of Brescia, Brescia, Italy; ^2^Section of Anatomy and Histology, Department of Neurosciences, Biomedicine and Movement Sciences, School of Medicine, University of Verona, Verona, Italy

**Keywords:** Parkinson’s disease, NF-κB/c-Rel, mouse model, non-motor symptoms, anxiety, depression, apathy

## Abstract

Non-motor symptoms are frequently observed in Parkinson’s disease (PD) and precede the onset of motor deficits by years. Among them, neuropsychiatric symptoms, including anxiety, depression, and apathy, are increasingly considered as a major challenge for patients with PD and their caregivers. We recently reported that mice lacking the nuclear factor-κB (NF-κB)/c-Rel protein (c-rel^–/–^ mice) develop an age-dependent PD-like pathology and phenotype characterized by the onset of non-motor symptoms, including constipation and hyposmia, starting at 2 months of age, and motor deficits at 18 months. To assess whether c-rel^–/–^ mice also suffer from neuropsychiatric symptoms, in this study we tested different cohorts of wild-type (wt) and c-rel^–/–^ mice at 3, 6, 12, and 18–20 months with different behavioral tests. Mice lacking c-Rel displayed anxiety and depressive-like behavior starting in the premotor phase at 12 months, as indicated by the analysis with the open field (OF) test and the forced swim test with water wheel (FST), respectively. A deficit in the goal-oriented nesting building test was detected at 18–20 months, suggesting apathetic behavior. Taken together, these results indicate that c-rel^–/–^ mice recapitulate the onset and the progression of PD-related neuropsychiatric symptoms. Therefore, this animal model may represent a valuable tool to study the prodromal stage of PD and for testing new therapeutic strategies to alleviate neuropsychiatric symptoms.

## Introduction

Parkinson’s disease (PD) is clinically defined as a movement disorder characterized by the presence of motor symptoms, including bradykinesia, rigidity, tremor, and postural instability. In addition to highly invalidating motor manifestations, PD is also accompanied by a variety of non-motor symptoms, which frequently appear at the early stages or even before the motor phase of the disease (Kalia and Lang, 2015). These symptoms include constipation, impaired olfaction, pain, fatigue, sleep disturbance, urinary and sexual dysfunctions, and neuropsychiatric symptoms (Khoo et al., 2013; Titova et al., 2017).

The most common neuropsychiatric symptoms in PD are anxiety, depression, and apathy (Gallagher and Schrag, 2012; Starkstein et al., 2012; Tan, 2012; Aarsland and Kramberger, 2015; Castrioto et al., 2016; Cooney and Stacy, 2016). The neuropsychiatric disturbances are sometimes more distressing than motor disabilities and critically affect the disease management, adding an additional heavy burden to patients with PD and their families (Alvarado-Bolaños et al., 2015; Chahine et al., 2021).

Depression is a major determinant in reducing the quality of life of patients with PD, negatively impacting motor disabilities, cognitive impairment, and neuropsychiatric comorbidities (Marsh, 2013). Although the frequency of depression in PD varies in relation to population and symptom assessment, clinically relevant depressive symptoms may occur in 20–35% of patients with PD (Gallagher and Schrag, 2012; Starkstein et al., 2012; Tan, 2012; Aarsland and Kramberger, 2015; Castrioto et al., 2016; Cooney and Stacy, 2016). Once, depression in patients with PD was mainly considered as a reactive state to the perception of social disabilities associated with the disease progression; nowadays, depression is recognized to be caused by the disease itself. In support of this, depressive disorders in PD frequently develop in a range of time between 2 and 10 years before the onset of motor symptoms (Ishihara and Brayne, 2006; Pont-Sunyer et al., 2015; Schrag et al., 2015).

Anxiety disorders are more prevalent in patients with PD when compared with age-matched healthy controls, with a prevalence rating from 5.3 to 40% (Tan, 2012). The spectrum of anxiety disorders in PD include generalized anxiety disorder, panic attack, social phobia, agoraphobia, and obsessive-compulsive disorders (Tan, 2012; Chen and Marsh, 2014). Depression and anxiety often coexist in PD, with anxiety disorders occurring in up to 70% of patients with PD suffering from depression (Leentjens et al., 2011; Zhu et al., 2017). Like depression, anxiety was found to be more frequent during the premotor phase of patients with PD, occurring in up to 20 years before the onset of motor impairment (Shiba et al., 2000; Pont-Sunyer et al., 2015; Schrag et al., 2015).

Apathy is characterized by a reduced interest, motivation and participation in goal-oriented behavior, the lack of an initiative in initiating or completing activities, indifference, and flattened affect (Dujardin et al., 2007). As for other neuropsychiatric symptoms, the prevalence of apathy in PD depends on assessment methods and disease severity and has been reported to be between 16.5 and 70% (Gallagher and Schrag, 2012; Starkstein et al., 2012; Tan, 2012; Aarsland and Kramberger, 2015; Castrioto et al., 2016; Cooney and Stacy, 2016). In patients with PD, apathy has not only been diagnosed in the absence of neuropsychiatric symptoms but has also been associated with depression, anhedonia, and cognitive deficits (Gallagher and Schrag, 2012; Starkstein et al., 2012; Tan, 2012; Aarsland and Kramberger, 2015; Castrioto et al., 2016; Cooney and Stacy, 2016). It has been reported that apathy predates motor deficits, occurring more frequently during the 2-year premotor period (Pont-Sunyer et al., 2015).

Parkinson’s disease is pathologically characterized by the accumulation of insoluble α-synuclein fibrils and the loss of dopaminergic neurons in the nigrostriatal system. Several mechanisms have been proposed to contribute to PD α-synuclein aggregation and neurodegeneration, including mitochondrial deficits, oxidative stress, neuroinflammation, and impaired autophagy. Interestingly, these molecular pathways can be modulated by nuclear factor-κB (NF-κB), a ubiquitous transcription factor involved in inflammatory and immune responses (Lanzillotta et al., 2015; Bellucci et al., 2020). The NF-κB family consists of five different subunits (c-Rel, p65/RelA, p50, RelB, and p52), which combine to form transcriptionally active dimers (Gilmore and Wolenski, 2012). We recently showed that male mice deficient for NF-κB/c-Rel protein (c-rel^–/–^ mice) develop an age-dependent PD pathology and phenotype (Baiguera et al., 2012; Lanzillotta et al., 2015; Porrini et al., 2017; Parrella et al., 2019; Bellucci et al., 2020). In particular, the temporal and anatomical patterns of α-synuclein accumulation in c-rel^–/–^ mice, involving the enteric nervous system (ENS), the olfactory bulbs (OBs), the dorsal motor nucleus of the vagus (DMV), the locus coeruleus (LC), and substantia nigra pars compacta (SNpc), are in accordance with the disease staging developed by Braak, who correlates the diffusion of α-synuclein pathology in PD to the type and severity of the symptomatology (Del Tredici and Braak, 2016). In 2-month-old c-rel^–/–^ mice, α-synuclein is mildly accumulated in the myenteric ganglia of the distal colon (Parrella et al., 2019). From 5 months, the accumulation of α-synuclein is detectable in the DMV, LC, and OB (Parrella et al., 2019) and in the SNpc from 12 months (Baiguera et al., 2012; Parrella et al., 2019). At this age, α-synuclein pathology is associated with a drop of dopamine transporter (DAT) and vesicular monoamine transporter 2 (VMAT2) in the striatum, and a mild neuroinflammation state in the SNpc (Porrini et al., 2017; Parrella et al., 2019). Finally, at 18 months, c-rel^–/–^ mice exhibit the loss of nigral dopaminergic neurons and striatal dopaminergic terminals, as well as increased levels of microglial activity in the SNpc and striatum (Baiguera et al., 2012; Parrella et al., 2019).

The progression of the pathology in c-rel^–/–^ male mice was paralleled by the development of both non-motor and motor symptoms. Starting from early premotor stages (2 months of age), c-rel^–/–^ mice display intestinal constipation and hyposmia (Parrella et al., 2019), followed at 18 months by L-DOPA-reversible hypomotility and gait-related deficits (Baiguera et al., 2012). Moreover, our preliminary results suggested that c-rel^–/–^ mice could display increased anxiety levels with aging (Parrella et al., 2019).

In this study, we fully characterized neuropsychiatric symptoms in the c-rel^–/–^ mouse model. Different cohorts of wild-type (wt) and c-rel^–/–^ male mice in an age range spanning from 3 to 20 months have been tested for anxiety, depressive-like behavior, apathy, short-term memory, and fine motor function with a battery of behavioral tasks [including open field (OF) test, forced swim test with water wheel (FST), nest building test, Y-maze, novel object recognition (NOR) test, and adhesive removal test] widely used in rodent models of PD (Sedelis et al., 2001; Ho et al., 2011, 2014; Campos et al., 2013; Fleming et al., 2013; Paumier et al., 2013; Bonito-Oliva et al., 2014; Rial et al., 2014; Baumann et al., 2016; Kim et al., 2019; Yan et al., 2020).

## Materials and Methods

### Mice

C57BL/6 mice carrying the c-Rel gene null mutation (c-rel^–/–^) were generated by inserting the neomycin cassette into the fifth exon of the c-Rel gene (Liou et al., 1999). Both c-rel^–/–^ and c-rel^+/+^ wt mice were housed in the animal facility of the Department of Molecular and Translational Medicine of the University of Brescia, where they were maintained in individual ventilated cages under 12/12 h light/dark cycles with access to standard rodent food and water *ad libitum* (Baiguera et al., 2012; Parrella et al., 2019). Mice were housed in groups of 2–4/cage in cages provided with bedding bags containing corncob (Mucedola) as nesting material and enriched with red mouse houses (Tecniplast). Humidity and room temperature were maintained at 55% and 22–23°C, respectively. All animal studies were approved by the Animal Welfare Body of the University of Brescia (Organismo Preposto al Benessere degli Animali -OPBA-) and were in accordance with the Directive 2010/63/EU on the protection of animals used for scientific purposes. All the procedures performed accomplished the ethical standards of the University of Brescia. Sex differences have been reported in PD animal models and patients, with males suffering earlier and more severe symptoms (Vegeto et al., 2020). For these reasons, only male mice were used in this study.

### Behavioral Tests

Different cohorts of wt and c-rel^–/–^ mice at 3, 6, 12, and 18–20 months of age were tested with the behavioral tasks described below to assess anxiety, depressive-like behavior, apathy, short-term memory, and fine motor deficits. Every cohort of animals was analyzed once with only one behavioral test. The list of mice subjected to the different behavioral tests is reported in Table 1.

**TABLE 1:** List of mice/cages used in the behavioral tests.

		OF	FST	Nest building	Y-maze	NOR	AR
wt	3 months	12 animals	13 animals	6 cages			
	6 months	13 animals	19 animals	5 cages			
	12 months	13 animals	14 animals	5 cages			
	18–20 months	14 animals	15 animals	7 cages	18 animals	8 animals	7 animals
c-rel^–/–^	3 months	16 animals	16 animals	6 cages			
	6 months	13 animals	20 animals	4 cages			
	12 months	15 animals	14 animals	5 cages			
	18–20 months	16 animals	21 animals	10 cages	18 animals	10 animals	7 animals

*AR, Adhesive Removal; FST, Forced Swim Test with Water Wheel; NOR, Novel Object Recognition; OF, Open Field.*

#### Open Field Test

Open field test was used for the assessment of anxiety. Briefly, mice were placed in the center of a black plastic OF box [40 (length) × 40 (width) × 40 (height) cm] (Parrella et al., 2019) and allowed to freely explore the arena for 5 min. Mice behavior was recorded and analyzed by a video tracking system (Ugo Basile ANY-Maze). Anxiety status was analyzed by combining two different protocols in the same test (Simon et al., 1994; Bonito-Oliva et al., 2014). We monitored the time spent, the percentage of distance covered, and the average speed recorded in a central area of 800 cm^2^ (Bonito-Oliva et al., 2014). At the same time, thigmotaxis, the tendency to remain in a protected area (close to vertical surfaces), was investigated by monitoring the time spent, the percentage of distance covered, and the number of entries in a narrow peripheral area less than 2.5 cm away from the box walls (Simon et al., 1994). The percentage of distance traveled in the central and peripheral areas was assessed by the ratio between the distances covered in the mentioned areas to the total distance covered. Reduced visits to the central zone and a preference to stay close to the walls are associated with higher anxiety levels (Simon et al., 1994; Bonito-Oliva et al., 2014). The test was performed during the dark phase of the light-dark cycle.

#### Forced Swim Test With Water Wheel

Depression-like behavior was assessed with the FST (40803 model, Ugo Basile). The apparatus consists of a water tank [20 (length) × 8 (width) × 18 (height) cm] containing a small water wheel free to rotate and is based on the behavioral test for screening antidepressants developed by Porsolt et al. (1977), and modified by Nomura et al. (1982). During 5 min of the test, the mice were placed in the tank filled with warm water (25 ± 1°C), where they were forced to swim and from which they could not escape. After an initial period of vigorous swimming activity and escape attempts by climbing the wheel, the mice adopted a characteristic immobile posture, moving only when necessary to keep their heads above the water. The delay between the start of the test and the appearance of the first episode of immobility lasted for at least 1 s (latency to immobility), and the duration of the immobile position (immobility time) was scored by a researcher blind to mice identity (Castagné et al., 2011). The number of wheel rotations, turning time (i.e., the time spent by the mice turning the wheel), and maximum and average rpm (revolutions per min) were automatically recorded by a rotation decoder controlled by a software (Ugo Basile ANY-Maze). The task was performed during the light phase.

#### Nest Building Test

Nest building is a highly conserved behavior in rodents that can be used to investigate motivation and goal-orientation (Baumann et al., 2016; Yamada et al., 2018). The test was performed on groups of siblings housed together. Briefly, a small cellulose bag containing corncobs (Mucedola) was placed in an empty cage provided with standard food and water and housed 2–4 mice (Lijam et al., 1997). Every 30 min, the cage was monitored by a researcher blind to mice identity, and the time the mice needed to nibble the bag, remove the corncobs, and build the nest was recorded. The task was performed during the light phase, as previously reported in these nocturnal animals (Roper, 1975).

#### Y-Maze Test

Working memory was assessed using a Y-maze [arms 21 cm (long) by 4 cm (wide) with 40-cm walls] (Parrella et al., 2013). Briefly, the mouse was placed in one of the arms of the maze and allowed to freely explore the environment for 8 min. The mouse performance was recorded by a video tracking system (Ugo Basile ANY-Maze), and the total numbers of arm entries, the total distance traveled, and the arm choices were scored. An arm choice was defined as both forepaws and hind paws fully entering the arm. Spontaneous alternation behavior (SAB) score was calculated as the ratio of alternations (an arm choice differing from the previous two choices) to the total number of alternation opportunities. The task was performed during the light phase.

#### Novel Object Recognition Test

The novel object recognition test relies on the natural propensity of rodents to explore novel objects, and it has been used to assess short-term spatial memory (Grayson et al., 2015). The maze consists of a black plastic OF box [40 (length) × 40 (width) × 40 (height) cm]. The test is based on the protocol previously described (Parrella et al., 2013). Briefly, on the first day of the test (habituation day), the mouse was placed into the box and allowed to explore the field for 5 min. About 24 h later (test day), the habituated mouse was placed again into the box in the presence of two identical, non-toxic objects (laboratory flask caps) and was let to freely explore them for 5 min (trial 1). The mouse performance was recorded by a video tracking system (Ugo Basile ANY-Maze), and the time spent exploring the objects was scored, considering the exploration of any physical contact with an object and/or approach with obvious orientation to it within 5 cm. At the end of trial 1, the animal was returned to the home cage. After 3 min, the mouse was placed back into the arena where one of the familiar objects was replaced by a novel object (ping pong ball). The mouse was allowed to explore the arena for 5 min, and its behavior was recorded again by the video tracking system. Recognition index (RI) was calculated as the time spent by the animal exploring the novel object to the total time spent exploring both the objects. The task was performed during the light phase.

#### Adhesive Removal Test

Fine motor deficits were assessed by the adhesive removal test (Fleming et al., 2013). Briefly, the mouse was restrained by grasping its scruff, and one adhesive label was gently placed onto its snout by using a pair of small forceps. The time needed by the mouse to remove the adhesive was recorded. If the animal did not remove the label within 60 s, the trial ended and the adhesive was removed manually by the experimenter. Trials where the label fell off were not considered. We scored the best result over three trials. The task was performed during the light phase.

### Statistical Analysis

Statistical analysis was performed with the GraphPad Prism software. The value of *p* < 0.05 was considered significant. Comparisons between the two groups were performed using the parametric two-tailed unpaired Student’s *t*-test or the non-parametric Mann–Whitney test. Comparisons among more than two groups were performed using the parametric two-way ANOVA followed by Sidak’s multiple comparison test or the non-parametric Kruskal–Wallis test followed by Dunn’s multiple comparison test. The data analyzed using parametric tests were expressed as mean ± standard error of the mean (SEM), while the data analyzed using non-parametric tests were expressed as median ± interquartile range.

## Results

### c-rel^–/–^ Mice Display Age-Dependent Anxiety-Like Behavior

**FIGURE 1:**
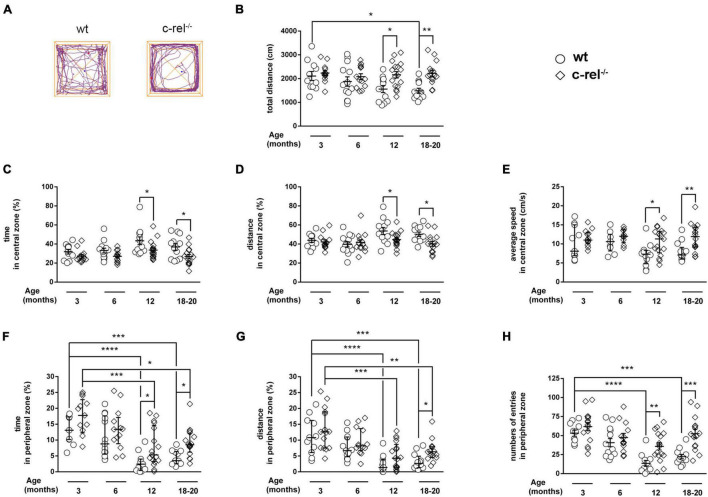
Different cohorts of wild-type (wt) and c-Rel protein (c-rel^–/–^) mice were tested for anxiety-like behavior in the open field (OF) at 3, 6, 12, and 18–20 months of age. **(A)** Representative track plots of 18–20-month-old wt and c-rel^–/–^ mice at the end of a 5-min OF test. The distance travelled by the mice is represented by the purple lines. The total distance covered **(B)**, the time spent, the distance covered, and the average speed calculated in the center of the apparatus (**C–E**, respectively), the time spent, the distance covered, and the number of entries registered in the peripheral area (**F–H**, respectively) are shown. No significant differences were found between wt and c-rel^–/–^ mice at 3 and 6 months in any of the described parameters. At 12 months, c-rel^–/–^ mice covered a higher total distance (**B**, *p* < 0.05), spent less time and walked a lower distance but at a higher speed in the central area (**C–E**, *p* < 0.05), and spent more time and entered more often in the peripheral area (**F**, *p* < 0.05 and **H**, *p* < 0.01, respectively). At 18–20 months, the differences between wt and c-rel^–/–^ mice became significant for the distance covered in the peripheral area (**G**, *p* < 0.05), remained significant for the time spent and distance covered in the central area, and the time spent in the peripheral area (**C,D,F**, *p* < 0.05), and further increased for the total distance covered (**B**, *p* < 0.001), the average speed in the central area (**E**, *p* < 0.01), and the number of entries in the peripheral area (**H**, *p* < 0.001). Moreover, the total distance traveled by wt mice, but not c-rel^–/–^ mice, decreased with aging (**B**: *p* < 0.05, wt 3 months vs. wt 18–10 months). Similarly, the number of entries in the peripheral area decreased with aging only in wt groups (**H**: *p* < 0.0001, wt 3 months vs. wt 12 months; *p* < 0.001, wt 3 months vs. wt 18–20 months). Finally, we observed an age-dependent decline in the time spent and the distance covered in the peripheral area for both wt and c-rel^–/–^ mice (**F,G**: *p* < 0.0001, wt 3 months vs. wt 12 months; *p* < 0.001, wt 3 months vs. wt 18–20 months; *p* < 0.001, c-rel^–/–^ 3 months vs. c-rel^–/–^ 12 months; *p* < 0.05, c-rel^–/–^ 3 months vs. c-rel^–/–^ 18–20 months). 3-month-old wt: 12 mice; 3-month-old c-rel^–/–^: 16 mice; 6-month-old wt: 13 mice; 6-month-old c-rel^–/–^: 13 mice; 12-month-old wt: 13 mice; 12-month-old c-rel^–/–^: 15 mice; 18–20-month-old wt: 14 mice; and 18–20-month-old c-rel^–/–^: 16 mice. *p < 0.05; **p < 0.01; ***p < 0.001, ****p < 0.0001. Two-way ANOVA followed by Sidak’s multiple comparison test in **(B–D,H)**; the Kruskal–Wallis test followed by Dunn’s multiple comparison test in **(E–G)**. Data are expressed as mean ± SEM in **(B–D,H)** or as median ± interquartile range in **(E–G)**.

Anxiety-like behavior was measured in the OF test. During the 5 min of the test, 12- and 18–20-month-old c-rel^–/–^ mice were significantly more active than wt, as indicated by the higher total distance covered (Figures 1A,B). Interestingly, in contrast to aged wt mice, 18–20-month-old c-rel^–/–^ mice did not show any age-dependent reduction in the total distance covered (Figure 1B). In these ages, c-rel^–/–^ mice spent less time in the center of the apparatus compared to wt mice (Figure 1C), in accordance with our previous findings (Parrella et al., 2019). Moreover, 12- and 18–20-month-old c-rel^–/–^ mice traveled a lower distance but at a higher speed in the central area (Figures 1D,E). In contrast, c-rel^–/–^ mice spent significantly more time and entered more often in the peripheral area at 12 and 18–20 months (Figures 1F,H). Similarly, 18–20-month-old c-rel^–/–^ mice covered a significantly higher distance in the peripheral zone (Figure 1G). Both the time spent and the distance traveled in the peripheral area by wt and c-rel^–/–^ mice decreased with aging, with wt animals showing a more marked decline (Figures 1F,G). Finally, we observed an age-dependent reduction of the number of entries in the peripheral area only in wt mice (Figure 1H).

### c-rel^–/–^ Mice Display Age-Dependent Depression-Like Behavior

**FIGURE 2:**
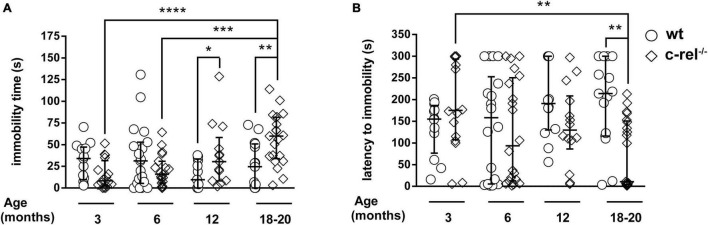
Different cohorts of wt and c-rel^–/–^ mice were tested for depression-like behavior with the FST at 3, 6, 12, and 18–20 months of age. The immobility time and latency to immobility are shown (**A,B**, respectively). A higher, age-dependent, immobility time was observed in 12-month-old (**A**: *p* < 0.05, wt 12 months vs. c-rel^–/–^ 12 months) and 18–20-month-old c-rel^–/–^ mice (**A**: *p* < 0.01, wt 18–20 months vs. c-rel^–/–^ 18–20 months; *p* < 0.0001, c-rel^–/–^ 3 months vs. c-rel^–/–^ 18–20 months; *p* < 0.001, c-rel^–/–^ 6 months vs. c-rel^–/–^ 18–20 months). Moreover, 18–20-month-old c-rel^–/–^ mice displayed a shorter, age-dependent, latency to immobility compared with age-matched wt mice (**B**, *p* < 0.01, wt 18–20 months vs. c-rel^–/–^ 18–20 months; *p* < 0.01, c-rel^–/–^ 3 months vs. c-rel^–/–^ 18–20 months). 3-month-old wt: 13 mice; 3-month-old c-rel^–/–^: 16 mice; 6-month-old wt: 19 mice; 6-month-old c-rel^–/–^: 20 mice; 12-month-old wt: 14 mice; 12-month-old c-rel^–/–^: 14 mice; 18–20-month-old wt: 15 mice; and 18–20-month-old c-rel^–/–^: 21 mice. *p < 0.05; **p < 0.01; ***p < 0.001; ****p < 0.0001. The Kruskal–Wallis test followed by Dunn’s multiple comparison test. Data are expressed as median ± interquartile range.

When tested with the FST, c-rel^–/–^ mice displayed an age-dependent increase of total immobility time at 12 and 18–20 months of age (Figure 2A). A similar age-dependent difference was observed to examine the latency to immobility, with c-rel^–/–^ mice showing the first episode of immobility significantly earlier than wt at 18–20 months (Figure 2B). In contrast, the number of wheel rotations, turning time, and maximum and average rpm did not vary between wt and c-rel^–/–^ groups in any of the considered ages (Supplementary Figures 1B–E). Body weight, a factor affecting the behavior of rodents in the FST (Bogdanova et al., 2013), did not differ between c-rel^–/–^ and wt mice in any of the considered ages (Supplementary Figure 1A).

### c-rel^–/–^ Mice Display Age-Dependent Apathetic Behavior

**FIGURE 3:**
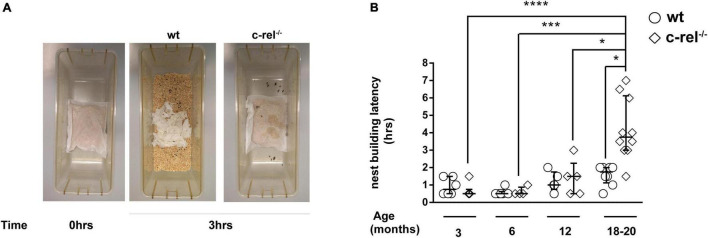
Different cohorts of wt and c-rel^–/–^ mice were tested for apathetic behavior with the nest building test at 3, 6, 12, and 18–20 months of age. Cages containing 2–4 mice per cage were used to evaluate the latency to build the nest. A small cellulose bag containing corncobs (Mucedola) was placed in the cage, and every 30 min, the nest building was monitored. **(A)** Representative images of 18–20-month-old wt and c-rel^–/–^ mice nest building at time 0 and after 3 h following the introduction of bags containing corncobs. **(B)** The latency to build the nest is shown. c-rel^–/–^ mice took a longer time to prepare the nest at 18–20 months of age (*p* < 0.05). Moreover, the nest building latency of 18–20-month-old c-rel^–/–^ mice was significantly higher than the values scored for c-rel^–/–^ mice at younger ages (*p* < 0.0001, c-rel^–/–^ 3 months vs. c-rel^–/–^ 18–20 months; *p* < 0.001, c-rel^–/–^ 6 months vs. c-rel^–/–^ 18–20 months; *p* < 0.05 c-rel^–/–^ 12 months vs. c-rel^–/–^ 18–20 months). 3-month-old wt: 6 cages; 3-month-old c-rel^–/–^: 6 cages; 6-month-old wt: 5 cages; 6-month-old c-rel^–/–^: 4 cages; 12-month-old wt: 5 cages; 12-month-old c-rel^–/–^: 5 cages; 18–20-month-old wt: 7 cages; and 18–20-month-old c-rel^–/–^: 10 cages; *p < 0.05; ***p < 0.001; ****p < 0.0001. The Kruskal–Wallis test followed by Dunn’s multiple comparison test. Data are expressed as median ± interquartile range.

In the nest building test, 18–20-month-old c-rel^–/–^ mice took a longer time to open the cellulose bag and build a nest when compared to wt mice and to c-rel^–/–^ mice at younger ages (Figures 3A,B). Nest building performance has been shown to rely on several factors, including cognitive skills, in particular hippocampus function (Jirkof, 2014), and fine motor activities (Paumier et al., 2013).

**FIGURE 4:**
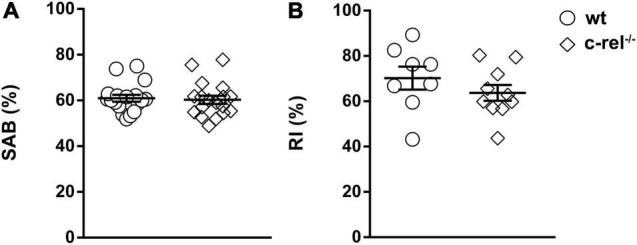
Two different cohorts of wt and c-rel^–/–^ male mice were tested for working and spatial short-term memory with Y-maze and novel object recognition (NOR) tests at 18–20 months of age. **(A)** In the Y-maze, spontaneous alternation behavior (SAB) percentage did not vary between wt and c-rel^–/–^ male mice. wt: 18 mice; c-rel^–/–^: 18 mice; *p* > 0.05, *t*-test. **(B)** On trial 2 of the NOR test, recognition index (RI) did not change between the groups. wt: 8 mice; c-rel^–/–^: 10 mice; *p* > 0.05, *t*-test. Data are expressed as mean ± SEM.

To investigate whether the poor nest building performance of c-rel^–/–^ mice was related to memory dysfunction, we tested 18–20-month-old wt and c-rel^–/–^ animals with Y-maze and NOR tests, two tasks used to analyze hippocampus-dependent working and short-term memory (Ho et al., 2011, 2014; Li et al., 2011; Grayson et al., 2015; Kim et al., 2019). In the Y-maze, alternation rates did not vary between the two groups (Figure 4A). Conversely, c-rel^–/–^ mice showed a significant increase in the total number of entries and total distance moved, two indicators of activity levels (Parrella et al., 2013; Supplementary Figures 2A,B). On trial 1 of the NOR test, the mice were allowed to explore a box containing two identical objects. As expected, no preference for one of the two objects was detected neither in wt nor in c-rel^–/–^ mice (Supplementary Figure 2C). At the end of the trial, the mice were returned to their home cages for 3 min and then placed again into the box where one of the objects was replaced with a novel one (trial 2). Both the mice groups recognized the novel object, as indicated by elevated and similar values of RI (Figure 4B). Taken together, these results indicate that aged c-rel^–/–^ mice did not suffer any impairment in working and short-term spatial memory.

Finally, we tested 18–20-month-old wt and c-rel^–/–^ animals with the adhesive removal test, a task used to evaluate fine motor function in rodents (Fleming et al., 2013). We did not find any significant difference in the time needed to remove the adhesive label between the two experimental groups (Supplementary Figure 3).

## Discussion

The results here reported point out clear age-dependent behavioral differences between wt and c-rel^–/–^ mice.

In the OF test, 12- and 18–20-month-old c-rel^–/–^ mice avoided the center of the field, and when they walked through the central area, they showed a higher locomotion speed compared with wt mice. Conversely, 12- and 18–20-month-old c-rel^–/–^ mice preferred to stay close to the arena walls. Interestingly, wt mice displayed an age-dependent decline of the total distance covered, of the time spent, of the distance covered, and of the number of entries in the peripheral area. In contrast, in c-rel^–/–^ mice, the age-dependent reduction of these parameters was milder or absent. In the OF test, avoiding open spaces and thigmotaxis are generally interpreted as anxiety-like behavior (Simon et al., 1994; Bonito-Oliva et al., 2014). During the 5 min of the OF test, 12-month-old c-rel^–/–^ mice were significantly more active than wt, and the difference between the two groups further increased at 18–20 months. Interestingly, hyperactivity of aged c-rel^–/–^ mice was confirmed in the Y-maze test, where c-rel^–/–^ mice showed a higher number of arm entries and traveled a longer distance than wt controls. Of note, this phenomenon was observed in both dark and light phases (in the OF and Y-maze, respectively). Indeed, hyperactivity is a behavior observed in different PD animal models, including LRRK2 (Volta et al., 2015; Zhang et al., 2021) and α-synuclein transgenic rodents (Unger et al., 2006; Graham and Sidhu, 2010; Polissidis et al., 2021). Moreover, hyperactivity has been related to psychosis-like behavior in rodents (Polissidis et al., 2021). Psychosis is a neuropsychiatric symptom occurring in PD as complication of dopamine therapy, but also manifesting in a premotor phase of the disease in the absence of PD medications (Ravina et al., 2007; Pagonabarraga et al., 2016). Future studies will further investigate psychosis-like behavior in c-rel^–/–^ mice. Of note, c-rel^–/–^ mice displayed overt behavioral abnormalities in the OF starting at 12 months but not at younger ages. This is in line with previous findings indicating similar levels of basal anxiety levels and locomotor activity between young c-rel^–/–^ and wt mice in the OF task (Ahn et al., 2008).

When tested with FST, 12- and 18–20-month-old c-rel^–/–^ mice displayed increased immobility time and decreased latency to immobility, two standard behavioral paradigms indicative of depression (Castagné et al., 2011; Bogdanova et al., 2013). These findings are in accordance with the previous results obtained with other rodent PD models (Campos et al., 2013; Baumann et al., 2016; Kim et al., 2019). On the other hand, neither did we detect any difference in the wheel rotation paradigm between c-rel^–/–^ and wt mice nor did we find a correlation between swimming activity and escape attempts by rotating the wheel. This discrepancy may be caused by the experimental protocol adopted; we cannot exclude that a different protocol, including a habituation trial, in which the rodent is left to explore the tank to identify the wheel as a possible escape way, will provide different results. The performance of c-rel^–/–^ mice in the FST could have been influenced at late age by motor deficits, that we described appearing at 18 months (Baiguera et al., 2012). However, a finding that aged c-rel^–/–^ mice turned the wheel vigorously as wt points out only a mild motor impairment of the PD model between 12 and 20 months. This finding supports the hypothesis that depressive-like behavior may play a role in the abnormal behavior observed in this test.

We found that c-rel^–/–^ mice exhibited a significant decrease in nesting behavior at 18–20 months. Nesting behavior is driven by several factors, including cognition, fine orofacial and forepaw motor functions, and emotional state (Sedelis et al., 2001; Paumier et al., 2013; Baumann et al., 2016; Kim et al., 2019). c-Rel plays a pivotal role in synaptic plasticity and long-term memory formation (Levenson et al., 2004; O’Riordan et al., 2006; Ahn et al., 2008). In confirmation of this, young c-rel^–/–^ mice displayed specific deficits in hippocampus-dependent long-term memory (Levenson et al., 2004; O’Riordan et al., 2006; Ahn et al., 2008). Nevertheless, the early long-term memory deficit does not hamper the capability of c-rel^–/–^ mice to build the nest at young ages. On the other hand, short-term memory is intact in both young (Ahn et al., 2008) and aged c-rel^–/–^ mice as shown in the case of Y-maze and NOR tests. Taken together, these findings suggest that cognition is not involved in the nesting deficit observed in c-rel^–/–^ mice. When tested with the adhesive removal test, aged c-rel^–/–^ mice took approximately 60% more time than wt mice to remove the adhesive label, albeit no significant difference was found between the two groups. This is in line with our previous gaiting analysis, indicating a mild reduction of forelimb print length in aged c-rel^–/–^ mice that may be related to muscle tone rigidity (Baiguera et al., 2012). Conversely, the time needed by aged c-rel^–/–^ mice to build the nest was almost 300% longer than that of controls. Although we cannot completely exclude the involvement of fine motor impairment in this abnormal behavior, taken together these findings suggest a strong emotional component in the nesting deficit. Interestingly, nesting behavior may mirror the “activity of daily living” in humans (Jirkof, 2014), and its impairment in PD rodent models has been associated with apathy and dysfunction in motivation that can be found in patients with PD (Sedelis et al., 2001; Paumier et al., 2013; Baumann et al., 2016; Kim et al., 2019).

Several factors can underlie the abnormal behavior observed in c-rel^–/–^ mice. For example, hyposmia, a deficit that affects c-rel^–/–^ mice starting at 2 months and that gets worse with aging (Parrella et al., 2019), can contribute to these alterations. In support of this, some of the detected behavioral abnormalities, such as hyperactivity and increased exploratory behavior, are consistently observed in animals subjected to olfactory bulbectomy, a model of depression (Song and Leonard, 2005; Zueger et al., 2005). Similarly, the genetic ablation of an olfactory function promoted anxiety-like behavior in mice (Glinka et al., 2012). Furthermore, bilateral transections of the lateral olfactory tract cause nest building deficits in hamsters (Marques et al., 1982).

The hyperactivity of c-rel^–/–^ mice may be driven by an anxiety status in response to a novel environment (Nakajima et al., 2020). Moreover, hyperactivity was observed in knockout rats for the gene encoding DAT (Leo et al., 2018). Hyperactivity in c-rel^–/–^ male mice was detectable starting at 12 months, an age at which a drop in striatal levels of DAT was observed (Parrella et al., 2019). These findings suggest a possible role of dopamine and DAT dysfunctions in the observed hyperactivity.

VMAT2 LO mice, a PD animal model lacking VMAT2, the monoamine transporter that we found to be reduced in the striatum of 12-month-old c-rel^–/–^ mice (Parrella et al., 2019), show an age-dependent apathetic behavior in the nest building test similar to that observed in our study (Baumann et al., 2016).

LC, the major norepinephrine-producing nucleus in the brain, plays a crucial role in mood regulation (Ressler and Nemeroff, 1999). The early and progressive deposition of fibrillary α-synuclein in the LC of c-rel^–/–^ mice may be strongly involved in the detected abnormal behavior (Parrella et al., 2019). In line with our mouse model, transgenic mice expressing human α-synuclein in LC neurons display an age-dependent anxiety-like behavior (Butkovich et al., 2020). Importantly, a potential role for α-synuclein pathology and degeneration occurring in the LC has been suggested for PD neuropsychiatric symptoms such as anxiety and depression (Butkovich et al., 2018; Weinshenker, 2018). Interestingly, the temporal pattern of the LC α-synuclein pathology in c-rel^–/–^ mice, starting at 5 months, correlates with the appearance of anxiety and depressive symptoms, appearing between 6 and 12 months.

## Conclusion

The present study shows that a variety of neuropsychiatric symptoms associated with PD, including anxiety, depressive-like behavior, and apathy, are reproduced in the c-rel^–/–^ mice. This animal model, by mimicking the premotor phase and cardinal histopathological features of PD, may contribute to the research on the pathophysiology of PD prodromal stage and to the development of new therapeutic strategies (Cerri and Blandini, 2020).

## Data Availability Statement

The datasets and materials used and/or analyzed during the current study are available from the corresponding author on reasonable request.

## Ethics Statement

All animal studies were reviewed and approved by the Animal Welfare Body of the University of Brescia (Organismo Preposto al Benessere degli Animali, OPBA) and were in accordance with the Directive 2010/63/EU on the protection of animals used for scientific purposes.

## Author Contributions

EP conceptualized the study, managed the mouse colony, designed and performed the behavioral studies, performed the data analysis, and wrote the manuscript. FDG designed and performed the behavioral studies, performed the data analysis, and edited the manuscript. VP prepared the figures and edited the manuscript. CG and PFF edited the manuscript. MB contributed to animal care. MP supervised the study, provided funding, and edited the manuscript. All authors contributed to the article and approved the submitted version.

## Conflict of Interest

The authors declare that the research was conducted in the absence of any commercial or financial relationships that could be construed as a potential conflict of interest.

## Publisher’s Note

All claims expressed in this article are solely those of the authors and do not necessarily represent those of their affiliated organizations, or those of the publisher, the editors and the reviewers. Any product that may be evaluated in this article, or claim that may be made by its manufacturer, is not guaranteed or endorsed by the publisher.

## Supplementary Material

Supplementary Figure 1 Different cohorts of wild-type (wt) and c-Rel protein (c-rel^–/–^) male mice were tested for depression-like behavior with the FST at 3, 6, 12, and 18–20 months of age. Body weight, number of wheel rotations, turning time, maximum, and average rpm are shown (**A–E**, respectively). The scored parameters did not differ between c-rel^–/–^ and wt mice in any of the considered ages (**A–E**: *p* > 0.05). We observed an age-dependent increase of body weight in both wt and c-rel^–/–^ mice (**A**: *p* < 0.001, 12- vs. 3-month-old wt; *p* < 0.0001, 12-month-old c-rel^–/–^ vs. 3-month-old c-rel^–/–^; *p* < 0.01, 18–20-month-old wt vs. 3-month-old wt; and *p* < 0.0001, 18–20-month-old c-rel^–/–^ vs. 3-month-old c-rel^–/–^). Data are expressed as mean ± SEM 3-month-old wt: 13 mice; 3-month-old c-rel^–/–^: 16 mice; 6-month-old wt: 19 mice; 6-month-old c-rel^–/–^: 20 mice; 12-month-old wt: 14 mice; 12-month-old c-rel^–/–^: 14 mice; 18-month-old wt: 15 mice; and 18-month-old c-rel^–/–^: 21 mice. ***p* < 0.01; ****p* < 0.001; *****p* < 0.0001. Two-way ANOVA followed by Sidak’s multiple comparison test in **(A,D)**; the Kruskal–Wallis test followed by Dunn’s multiple comparison test in **(B,C,E)**. Data are expressed as mean ± SEM in **(A,D)**, or as median ± interquartile range in **(B,C,E)**.

Supplementary Figure 2 Two different cohorts of wt and c-rel^–/–^ male mice were tested with Y-maze and novel object recognition (NOR) tests at 18–20 months of age. **(A,B)** In the Y-maze, c-rel^–/–^ mice displayed a higher number of arm entries and traveled a longer distance than wt animals. Data are expressed as mean ± SEM wt: 18 mice; c-rel^–/–^: 18 mice. ^****^*p* < 0.0001, *t*-test. **(C)** On trial 1 of the NOR test, rodents were allowed to explore a box containing two identical objects (objects **A,B**), and the time spent exploring them was recorded. No significant difference was found in the time the animals dedicated to exploring the two objects. wt: 8 mice; c-rel^–/–^: 10 mice; *p* > 0.05, *t*-test. Data are expressed as mean ± SEM.

Supplementary Figure 3 In total, 18–20-month-old wt and c-rel^–/–^ male mice were tested with the adhesive removal test. No significant difference was found between the two groups in the time needed to remove the adhesive label. wt: 7 mice; c-rel^–/–^: 7 mice; *p* > 0.05, Mann–Whitney test. Data are expressed as median ± interquartile range.
