# Supplementary figures and images for: Age-Dependent Neuropsychiatric Symptoms in the NF-κB/c-Rel Knockout Mouse Model of Parkinson’s Disease
Source: Front Behav Neurosci. 2022 Mar 11;16:831664. doi: 10.3389/fnbeh.2022.831664 (PMC8965703; doi:10.3389/fnbeh.2022.831664)

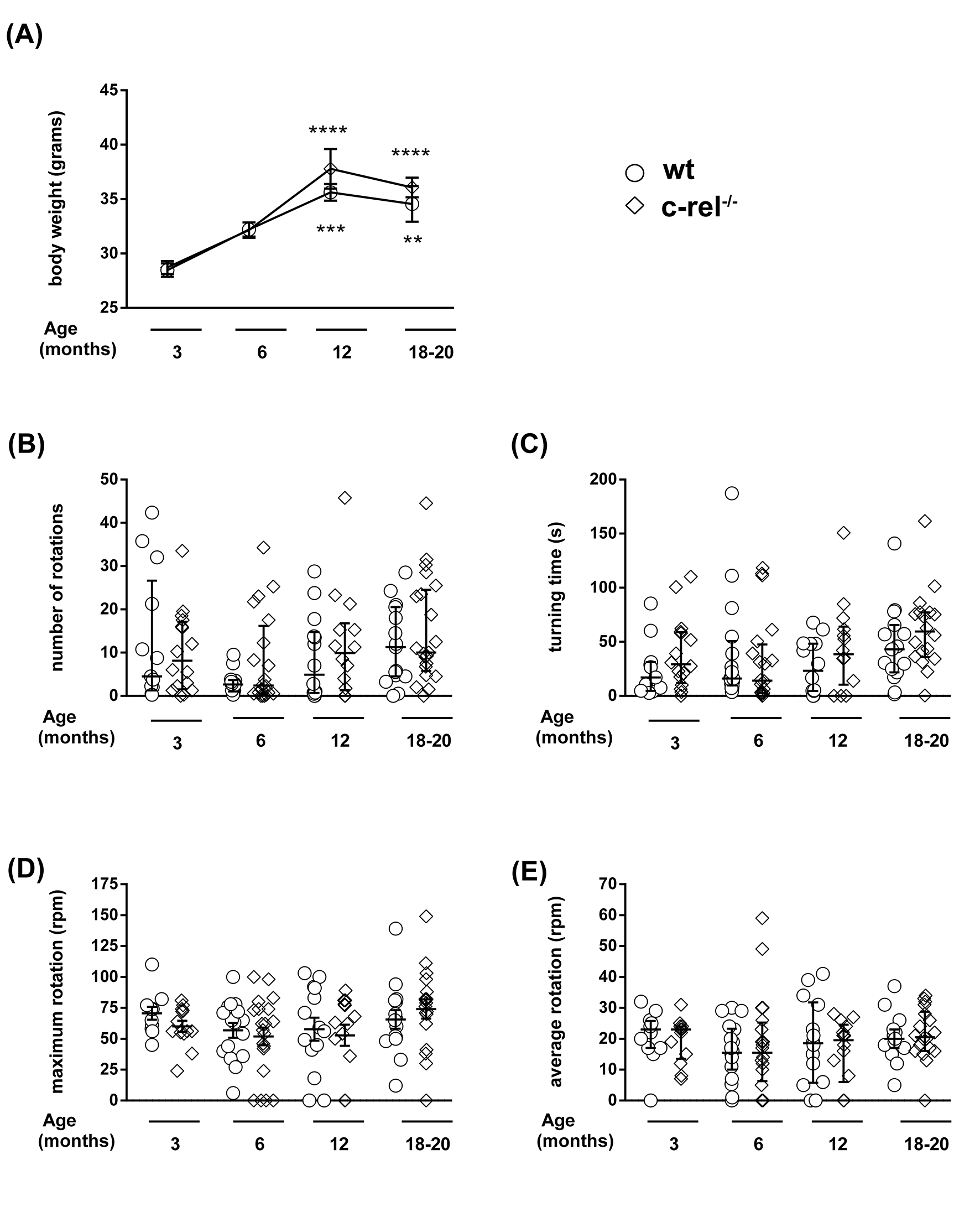

Supplement: Supplementary Figure 1 — Different cohorts of wild-type (wt) and c-Rel protein (c-rel–/–) male mice were tested for depression-like behavior with the FST at 3, 6, 12, and 18–20 months of age. Body weight, number of wheel rotations, turning time, maximum, and average rpm are shown (A–E, respectively). The scored parameters did not differ between c-rel–/– and wt mice in any of the considered ages (A–E: p > 0.05). We observed an age-dependent increase of body weight in both wt and c-rel–/– mice (A: p < 0.001, 12- vs. 3-month-old wt; p < 0.0001, 12-month-old c-rel–/– vs. 3-month-old c-rel–/–; p < 0.01, 18–20-month-old wt vs. 3-month-old wt; and p < 0.0001, 18–20-month-old c-rel–/– vs. 3-month-old c-rel–/–). Data are expressed as mean ± SEM 3-month-old wt: 13 mice; 3-month-old c-rel–/–: 16 mice; 6-month-old wt: 19 mice; 6-month-old c-rel–/–: 20 mice; 12-month-old wt: 14 mice; 12-month-old c-rel–/–: 14 mice; 18-month-old wt: 15 mice; and 18-month-old c-rel–/–: 21 mice. **p < 0.01; ***p < 0.001; ****p < 0.0001. Two-way ANOVA followed by Sidak’s multiple comparison test in (A,D); the Kruskal–Wallis test followed by Dunn’s multiple comparison test in (B,C,E). Data are expressed as mean ± SEM in (A,D), or as median ± interquartile range in (B,C,E). [file Image_1.TIF]

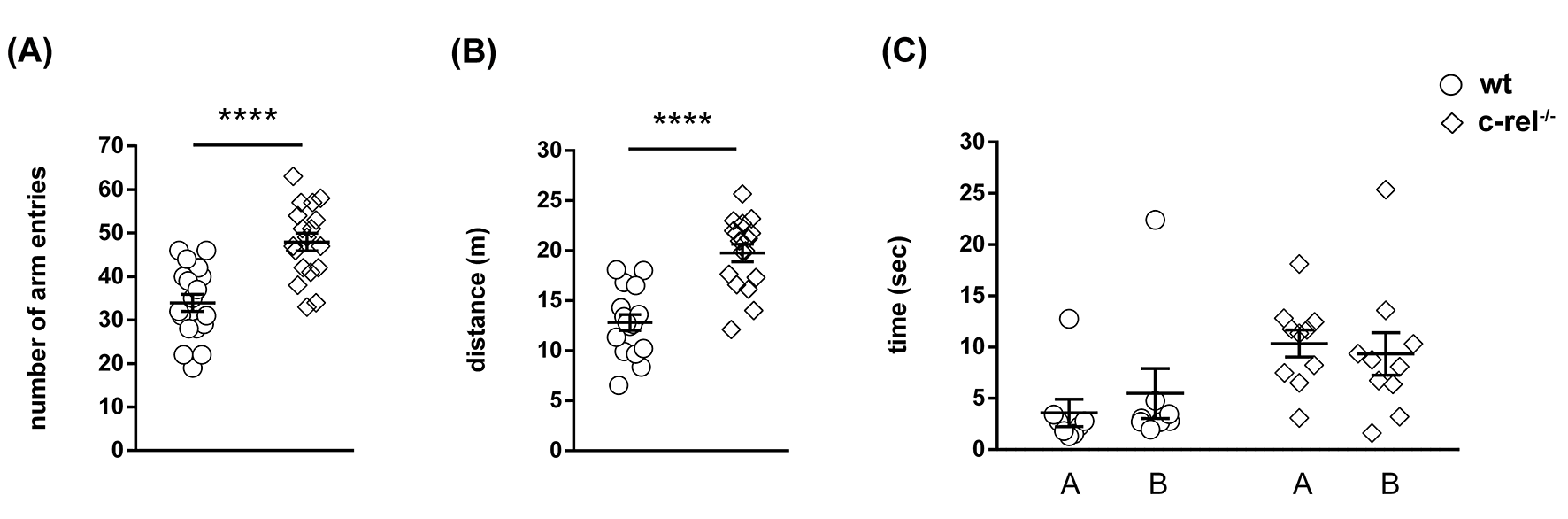

Supplement: Supplementary Figure 2 — Two different cohorts of wt and c-rel–/– male mice were tested with Y-maze and novel object recognition (NOR) tests at 18–20 months of age. (A,B) In the Y-maze, c-rel–/– mice displayed a higher number of arm entries and traveled a longer distance than wt animals. Data are expressed as mean ± SEM wt: 18 mice; c-rel–/–: 18 mice. ****p < 0.0001, t-test. (C) On trial 1 of the NOR test, rodents were allowed to explore a box containing two identical objects (objects A,B), and the time spent exploring them was recorded. No significant difference was found in the time the animals dedicated to exploring the two objects. wt: 8 mice; c-rel–/–: 10 mice; p > 0.05, t-test. Data are expressed as mean ± SEM. [file Image_2.TIF]

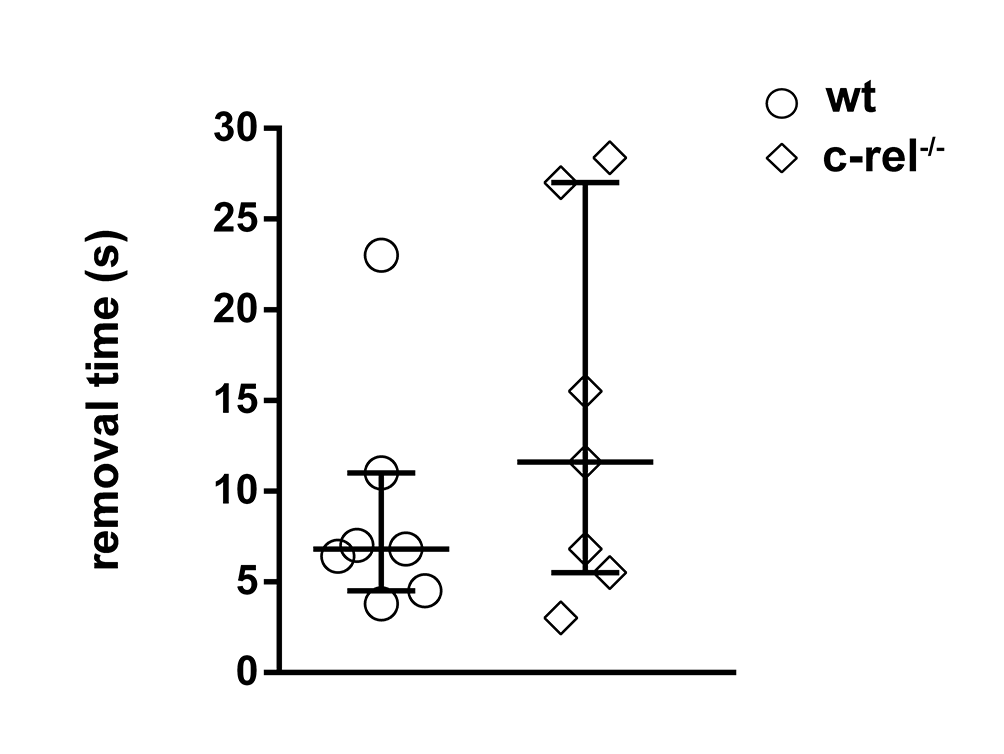

Supplement: Supplementary Figure 3 — In total, 18–20-month-old wt and c-rel–/– male mice were tested with the adhesive removal test. No significant difference was found between the two groups in the time needed to remove the adhesive label. wt: 7 mice; c-rel–/–: 7 mice; p > 0.05, Mann–Whitney test. Data are expressed as median ± interquartile range. [file Image_3.TIF]
